# Functionalities of *Tremella fuciformis* Polysaccharides Modified with Gallic Acid

**DOI:** 10.3390/molecules29245890

**Published:** 2024-12-13

**Authors:** Tai-Ti Liu, Kai-Siang Hong, Tsung-Shi Yang

**Affiliations:** 1 Department of Food Science, Yuanpei University of Medical Technology, No. 306 Yuanpei Street, Hsinchu 30015, Taiwan; taiti@mail.ypu.edu.tw (T.-T.L.); brian84729@gmail.com (K.-S.H.); 2 Department of Cosmeceutics, China Medical University, No. 100, Sec. 1, Jingmao Rd., Beitun Dist., Taichung 406040, Taiwan

**Keywords:** *Tremella fuciformis*, polysaccharide, gallic acid, antioxidant activity, zinc complex, antimicrobial activity

## Abstract

This research aimed to modify polysaccharides extracted from the edible mushroom *Tremella fuciformis* with gallic acid (GA) and to complex them with zinc ions. The functionalities of the modified *Tremella fuciformis* polysaccharides (TFPs) were investigated. Regarding antioxidant activity, TFP-GA demonstrated effective scavenging activity against DPPH radicals, nitric oxide, and hydrogen peroxide. Additionally, TFP-GA exhibited superior reducing ability toward Fe^3+^ and enhanced chelating activity toward Fe^2+^ compared to unmodified TFP. Notably, the TFP-GA conjugate outperformed GA in Fe^2+^-chelating activity. In terms of antimicrobial activity, the TFP-GA-Zn complex showed significantly improved antimicrobial effectiveness against *S. aureus* and *E. coli* compared to TFP-GA.

## 1. Introduction

*Tremella fuciformis* (TF) is a tropical and subtropical white jelly fungus, also known as snow fungus or silver ear, characterized by its color and appearance. TF is an edible mushroom widely utilized as an ingredient in both Chinese cuisine and traditional medicine. Polysaccharides are the major components of TF. The structure of TFP consists of α-D-mannose as the main backbone chain, with β-D-xylose, β-D-gluconic acid, and β-D-xylobiose linked to the C-2 position of the main chain mannose [1]. The structural characteristics and many biological functions of TFP such as anti-oxidation, anti-aging, anti-inflammation have been extensively reviewed and reported [2,3,4].

Although the antioxidant activity of TFP has been reported, the hydroxyl groups of polysaccharides are not good hydrogen atom or electron donors. Therefore, they are not effective antioxidants. The modification of polysaccharides can enhance their functionality. Low-molecular-weight TFP has been modified by sulfation, and the modified TFP exhibited stronger scavenging activity against superoxide anions, DPPH, and hydroxyl radicals compared to raw TFP [5]. Additionally, the antioxidant and moisture-preserving activities of TFP can be enhanced through carboxymethylation [6]. Due to their characteristic structure, phenolic compounds generally possess strong antioxidant abilities. They are often incorporated into the structures of other molecules, such as polysaccharides [7] and proteins [8], to provide new functionalities, including antioxidant activity. TFP has been modified with catechin using a free radical grafting method to enhance its antioxidant activity. However, this method may lead to the degradation of polysaccharides [9], which can reduce the viscosity of the resulting products. This degradation affects the rheological properties of the polysaccharides, particularly when they are used as texture modifiers or stabilizers in a product. Furthermore, the compositions of the chemical structures of the reacted products become more complex and might vary more between batches due to the combined effect of the random attack of radicals and the varied degree of degradation of the polysaccharides.

Optionally, the EDC (1-ethyl-3-(3-dimethylaminopropyl)carbodiimide) or EDC/NHS (N-hydroxysuccinimide) method has been widely used in the modification of proteins or polysaccharides by catalyzing the formation of amide or ester bonds between carboxylic groups and amine groups or hydroxyl groups. The EDC-mediated coupling method requires only mild reaction conditions and can be conducted in a wide range of solvents such as distilled water, aqueous ethanol, or even absolute ethanol. The significant advantage of this method is that it does not incorporate foreign structures into the crosslinked products [10]. Additionally, the coupling reagents and byproducts can be easily removed after the reaction, resulting in clean products. Thus, this method has been used to prepare food-grade crosslinked gelatin nanoparticles for Pickering emulsion stabilization [10]. The role of NHS is to stabilize the intermediate formed between EDC and a carboxylic compound during the reaction process [11]. GA, a trihydroxybenzoic acid found abundantly in plant metabolites across the plant kingdom, possesses strong antioxidant and free radical scavenging properties. It effectively shields biological cells, tissues, and organs from damage caused by oxidative stress [12]. GA has been grafted onto collagen to enhance its structural stability using the EDC/NHS method [13]. Chitosan was conjugated with GA to improve its water solubility and enhance its antioxidant activity using a similar method [14]. Similarly, GA was grafted onto oat β-glucan and hydrolyzed oat β-glucan using the EDC/NHS method to enhance their antioxidant and bacteriostatic activities compared to the unmodified forms [15].

Zinc is an essential trace element widely present in subcellular metabolism and serves as an essential component of the catalytic site of at least one enzyme in every enzyme classification [16]. Polysaccharides have been used as a platform for complexing zinc ions, which could provide a better function than inorganic and organic zinc supplements in treating zinc deficiency and improving antioxidant activities in vivo [17]. The complex of zinc and polysaccharides from *Fritillaria ussuriensis* has been reported to enhance the scavenging ability of the polysaccharides against hydroxyl and superoxide anion radicals [18]. Apart from its role as an essential nutrient, zinc exhibits antimicrobial activity. Chondroitin sulfate complexed with zinc was found to have antibacterial and anti-inflammatory effects [19]. In the present study, GA was grafted onto TFP using the EDC/NHS method to covalently link the carboxylic groups of GA with the hydroxyl groups of TFP. The reactive site is not on the main chain of the polysaccharides; therefore, this reaction would not cause the degradation of TFP. Additionally, this grafting process was followed by complexation with zinc ions. The antioxidant and antimicrobial activities of the synthesized compounds were investigated.

## 2. Results and Discussion

### 2.1. Composition and Yield of TF Extracts

Thermal extraction of TF was performed using neutral and alkaline aqueous solutions. The composition and yield of the extracts are detailed in Table 1. Sugars, with polysaccharides as the main constituents of the extract, account for over 80% of the total. Neutral water extraction resulted in lower concentrations of sugars and overall yield compared to alkaline extraction. Conversely, protein and polyphenol levels were significantly higher in the neutral water extract. The alkaline method effectively breaks down plant cell walls, facilitating the extraction of polysaccharides. Additionally, acidic polysaccharides are more soluble under alkaline conditions [20]. Therefore, the alkaline method was favorably utilized to extract acidic TFP. The total polyphenol content was minimal at 0.1%, which was insufficient to significantly contribute to the antioxidant activity of TFP. Consequently, the antioxidant activity of TFP was further enhanced by modifying its structure with GA.

### 2.2. Synthesis of TFP-GA

GA was grafted onto TFP via the EDC/NHS-catalyzed method. Reaction conditions, yield, and grafting ratios for the synthesis of TFP-GA are shown in Table 2. The amounts of TFP and EDC were fixed, while the other reactants varied. The grafting ratio of GA was used to denote the grafting efficiency. When the amount of NHS was increased to a 1:1 ratio with EDC, keeping other factors consistent, the yield and grafting ratio of TFP-GA-5 were slightly lower than those of TFP-GA-1. This suggests that a higher NHS concentration did not further enhance the grafting efficiency. It appears that a higher proportion of EDC relative to NHS was more favorable for this reaction, a finding consistent with some studies on conjugation reactions [21,22].

Regarding pH effects, varying pH from 4.5 to 7 increased the grafting ratio from 2% to 6.3%, and the yield from 32% to 40%. This indicates that a higher pH was more beneficial for synthesizing TFP-GA. This can be attributed to the pKa of GA being 4.41 [23]; as pH increases, GA tends to form carboxylate anions, which couple more efficiently than carboxyl groups during the reaction [24].

Concerning the amount of GA used, increasing GA led to higher grafting ratios of TFP-GA. For instance, the grafting ratio of TFP-GA-7 (8.8%) was higher than that of TFP-GA-1 (6.3%); however, the yield showed the opposite trend, with values of 26.8% and 40% for TFP-GA-7 and TFP-GA-1, respectively. While more GA favored the grafting ratio of TFP-GA, the higher loss of unreacted GA during dialysis reduced the overall yield.

### 2.3. NMR Analysis of TFP-GA

**Figure 1:**
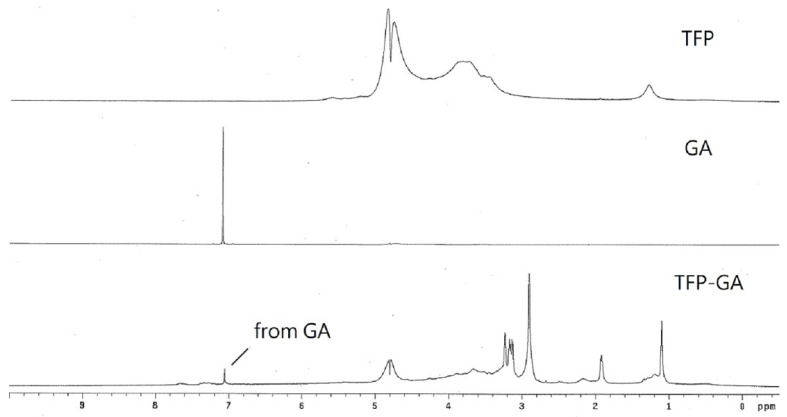
NMR spectra of TFP, GA, and TFP-GA.

The NMR spectra of TFP, GA, and TFP-GA are shown in Figure 1. In the spectrum of TFP-GA, a new peak appears at approximately 7.2 ppm, which is not present in the spectrum of TFP. This peak corresponds to the phenol group of GA, as observed in the spectra of GA and TFP. This indicates that GA has been chemically bonded to TFP.

### 2.4. Antioxidant Activity

#### 2.4.1. DPPH Assay

The DPPH assay is a fundamental and widely used method for assessing the antioxidant capacity of substances. DPPH is a stable free radical that changes color when it reacts with antioxidants. By measuring the degree of color change, the IC_50_ of the antioxidants can be determined. After calculating the relative reduction in IC_50_ values, it was found that the antioxidant activity of the synthesized compound increased 6-fold from TFP-GA-4 to TFP-GA-1, and 9.8-fold from TFP-GA-4 to TFP-GA-7 (Table 2). The grafting ratio of TFP-GA increased by 3.2 times from TFP-GA-4 to TFP-GA-1, and by 4.4 times from TFP-GA-4 to TFP-GA-7 (Table 1). Consequently, antioxidant activity increased with a higher grafting ratio of TFP-GA. When the fold increase in antioxidant activity was divided by the fold increase in the grafting ratio, the resulting values were 2.2 for TFP-GA-7 and 1.9 for TFP-GA-1 compared to TFP-GA-4. This indicates that grafting more GA onto TFP leads to more efficient antioxidant activity. However, there was a 33% decrease in yield from TFP-GA-1 to TFP-GA-7, which may compromise the efficiency of antioxidant activity gains by excessively increasing the GA content in the compound. Additionally, when TFP-GA was complexed with Zn^2+^, its antioxidant activity decreased by about 27%.

#### 2.4.2. Nitric Oxide Scavenging Activity

Nitric oxide is a reactive molecule produced from L-arginine by nitric oxide synthase and plays crucial roles in various physiological processes. However, excessive nitric oxide production can contribute to acute and chronic inflammation, posing a risk to affected tissues [25]. Consequently, inhibiting nitric oxide production may provide a therapeutic benefit by mitigating inflammation. In this study, we compared the antioxidant activity of synthesized compounds by evaluating their IC_50_ values for nitric oxide and DPPH radical scavenging. The results demonstrated that the increase in antioxidant activity from TFP-GA-4 to TFP-GA-1 was 8.7-fold, and from TFP-GA-4 to TFP-GA-7 was 19.3-fold. Notably, these values were significantly higher compared to their respective performances in the DPPH assay. This suggests that the increased grafting ratio of TFP-GA more effectively quenches nitric oxide radicals than DPPH radicals. Due to the high efficacy of the quenching effect, this activity was profoundly affected when TFP-GA was complexed with Zn^2+^, with its effectiveness decreasing to approximately 2 times its original value, indicating a significant reduction in activity.

#### 2.4.3. Hydrogen Peroxide Scavenging Activity

H_2_O_2_ is a significant reactive oxygen species in biological systems and its excess can damage cells and tissues. It tends to react poorly or not at all with most biological molecules. This is due to the high activation energy barrier that must be overcome to unleash its oxidizing capabilities. The O-O bond in hydrogen peroxide is relatively weak and can easily break apart when the substance is exposed to heat, radiation, light, or redox-active metals [26]. This breakdown produces hydroxyl radicals or, in some cases, leads to the formation of higher oxidation states of the metals. These secondary products are responsible for many of hydrogen peroxide’s potent oxidizing effects. The results in Table 3 showed that the IC_50_ value of TFP was not detected within the tested concentration range. However, TFP-GA exhibited H_2_O_2_ scavenging activity, which can be attributed to GA. This activity increased with the GA content in the conjugate. The ability of antioxidants to scavenge H_2_O_2_ can be linked to their capacity to donate electrons. Owing to the relatively poor reactivity of H_2_O_2_ with most molecules, as mentioned earlier, the IC_50_ values of the TFP-GA conjugates for scavenging H_2_O_2_ were higher than those for DPPH and NO radicals. In fact, the values for TFP-GA-3 and TFP-GA-4 could not be obtained within the tested concentration range. Similarly, both compounds demonstrated lower reducing power toward ferric ions. Regarding the complexation effect of Zn^2+^, there was a decrease of about 24% in the H_2_O_2_ scavenging activity of the TFP-GA-Zn complex.

#### 2.4.4. Ferric Ion Reducing Power

Phenolic acids can scavenge free radicals through three competitive mechanisms, which are influenced by the reaction conditions. These mechanisms include hydrogen atom transfer, single-electron transfer followed by proton transfer, and sequential proton loss followed by electron transfer. The latter is particularly favorable in aqueous solutions [27]. Therefore, the electron transfer ability, or reducing power, of GA is crucial in this study. To assess this, the ferric ion reducing power was measured as equivalents of ascorbic acid or GA. From Table 3, it is clear that TFP did not show any electron-reducing effect, but GA did. This also demonstrates that GA is the key compound responsible for this type of antioxidant activity. The reducing power of TFP-GA improved with an increase in the grafted GA content. The ferric ion reducing ability also increased with higher grafted GA levels. Furthermore, the reducing power decreased by approximately 15% when GT-GA was complexed with Zn^2+^.

#### 2.4.5. Fe^2+^-Chelating Ability

Reducing ability is a key mechanism by which antioxidants neutralize free radicals through electron transfer. However, certain metal ions, such as Fe^3+^, can be reduced to Fe^2+^, which may catalyze the generation of highly reactive hydroxyl radicals from H_2_O_2_, potentially exacerbating oxidative damage. Therefore, in addition to reducing ability, metal-ion chelating ability is an important attribute of effective antioxidants. In this study, neither TFP nor GA demonstrated Fe^2+^-chelating ability within the tested concentration range. However, when GA was grafted onto TFP, the resulting TFP-GA conjugate exhibited Fe^2+^-chelating capability, which increased with the GA content (Table 3). The metal chelating ability of polyphenols is often attributed to ortho-dihydroxy groups, such as catechol or galloyl groups [28]. For instance, the rhamnosyl glucoside derivative of quercetin (rutin) showed superior Fe^2+^-chelating ability compared to quercetin alone, suggesting that the glucoside moiety enhances the chelating potential of quercetin [29]. Chlorogenic acid, an ester of caffeic acid and quinic acid, exhibits metal chelating ability due to its catechol moiety. Although the adjacent 4,5-dihydroxy groups in the quinic acid ring are not in a planar conformation and thus cannot form a stable chelated complex on their own, chlorogenic acid demonstrates stronger Fe^2+^-chelating ability than caffeic acid. This enhancement might result from interactions between the dihydroxy groups of quinic acid and the catechol moiety of caffeic acid, either within the same molecule or in a different one. Similarly, GA alone did not show significant Fe^2+^-chelating ability, but this property was observed when GA was conjugated with chitosan [30] or sodium caseinate [31]. TFP-GA-Zn exhibited a lower Fe^2+^-chelating ability than TFP-GA, decreasing by about 70%. This may be due to the competition between Zn^2+^ and Fe^2+^, as both are cations that bind to the negatively charged sites of TFP-GA, significantly affecting this activity.

### 2.5. Antimicrobial Activity of TFP-GA Complexed with Zinc Ions

**Figure 2:**
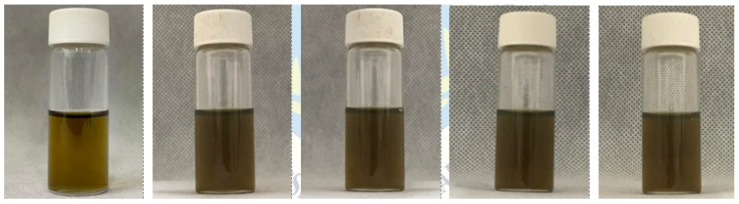
TFP-GA-Zn solutions prepared at different concentrations of zinc nitrate, from left to right: 0, 80, 120, 160, and 200 mM.

Polysaccharides can serve as platforms for metal incorporation to produce new materials with antibacterial activities. In this study, TFP-GA was complexed with Zn^2+^ to form TFP-GA-Zn. The zinc concentrations used are shown in Figure 2. No precipitation was observed up to 200 ppm; therefore, this concentration was selected for the antimicrobial activity test. TFP and TFP-GA did not show antimicrobial activity within the tested concentration range; however, TFP-GA-Zn exhibited an antimicrobial effect (Table 4). This activity is attributed to the presence of Zn^2+^ ions. TFP-GA-Zn demonstrated a stronger antimicrobial effect against *E. coli* compared to *S. aureus*, which differs from findings in some studies in the literature. The general trend observed in many studies is that Gram-positive bacteria, such as *S. aureus*, are often more sensitive to zinc compounds than Gram-negative bacteria like *E. coli* [32,33]. This sensitivity may be attributed to differences in cell wall structure and permeability. Specifically, Gram-positive bacteria have a thicker peptidoglycan layer, which might make them more susceptible to certain metal ions. In contrast, Gram-negative bacteria possess an outer membrane that can act as a barrier. The antimicrobial mechanisms of zinc include disrupting bacterial enzyme systems, interfering with protein synthesis, and damaging cellular membranes [34]. These mechanisms might differ between Gram-positive and Gram-negative bacteria, potentially influencing their relative sensitivity to zinc. However, some studies have reported no significant difference in zinc sensitivity between *S. aureus* and *E. coli* [35], or even greater susceptibility of *E. coli* under certain conditions [36]. Such discrepancies can arise from various factors, including the specific zinc compounds used, their concentrations, experimental conditions, and strain variations within each bacterial species [36].

## 3. Materials and Methods

### 3.1. Materials

TF was purchased from a Chinese medicinal herbs store in Taichung, Taiwan. GA, ascorbic acid, 2,2-diphenyl-1-picrylhydrazyl (DPPH), Folin–Ciocalteu’s phenol reagent, bicinchoninic acid (BCA), glucose, and zinc nitrate hexahydrate were obtained from Sigma-Aldrich Co. (St. Louis, MO, USA). Ferrozine, EDC hydrochloride, and NHS were purchased from Alfa Aesar Co. (Ward Hill, MA, USA). Ferric chloride hexahydrate was acquired from Merck (Darmstadt, Germany). Phenol was purchased from Shimakyu Company Limited (Samut Sakhon, Thailand). Potassium ferricyanide and trichloroacetic acid were obtained from Ferak Berlin GmbH (Berlin, Germany). Ferrous chloride tetrahydrate was sourced from J.T. Baker (Center Valley, PA, USA). Nutrient broth and agar were acquired from Difco Laboratories (Detroit, FL, USA).

### 3.2. Microbial Strains

*Staphylococcus aureus* (BCRC 10781) and *Escherichia coli* (BCRC 14824) were purchased from the Bioresource Collection and Research Center (BCRC) at the Food Industry Research and Development Institute in Hsinchu, Taiwan.

### 3.3. Extraction of TFP

Twenty grams of dried TF was ground to a fine powder. The powdered TF was mixed with 100 mL of 95% ethanol in a 1:5 ratio and stirred overnight to remove color materials. The ethanol was subsequently removed by centrifugation. Following the ethanol extraction, the residue was subjected to water extraction. Two hundred milliliters of deionized water was added to the residue at a 1:10 ratio. The mixture was incubated in a water bath at 80 °C for 2 h to facilitate comprehensive hydration and extraction. After incubation, the mixture was cooled to room temperature and then centrifuged at 8000 rpm for 30 min at 4 °C. The supernatant was collected for further processing. To precipitate the extracted components from the supernatant, ethanol was added to achieve a final concentration of 70%. A white precipitate formed and was collected by centrifugation at 5000 rpm for 10 min. The precipitate was dissolved in water and freeze-dried to yield a freeze-dried product designated as TFP-A. The remaining residue after water extraction underwent alkaline extraction. This residue was treated with 1 M NaOH at a 1:40 ratio and heated in a water bath at 65 °C for 2 h. Following the alkaline extraction, the resulting mixture was centrifuged at 8000 rpm for 30 min at 4 °C. The supernatant was collected and precipitated with ethanol using the same procedure as described above. The precipitate was re-dissolved in water and transferred to a dialysis tube (MWCO 7–8 kDa) for dialysis. Dialysis was performed over 2 d at 4 °C, using a total volume of 16 L of double-deionized water, with 4 L replaced periodically. The pH of the water was carefully monitored to ensure it matched that of the fresh water initially used, and the process was stopped once the pH had equilibrated. The extract was then freeze-dried, resulting in another freeze-dried product designated as TFP-B.

### 3.4. Analysis of Total Carbohydrate Content of TF Extract

The total carbohydrate content was measured using the phenol-sulfuric acid method [37] with modification. To prepare the polysaccharide solutions of TFP-A and TFP-B at 0.01% (*w*/*v*), 1 mL of each polysaccharide solution was mixed with 0.5 mL of 5% phenol solution. Next, 2.5 mL of concentrated sulfuric acid was added slowly, and the mixture was thoroughly mixed. After cooling in an ice bath for 30 min, 1 mL of the solution was transferred to a cuvette. The absorbance of polysaccharides A and B was measured at 490 nm using a spectrophotometer (Genesys G-10, Thermo Fisher Scientific, Waltham, MA, USA). A calibration curve was constructed using glucose as the standard. The absorbance values obtained were then substituted into the calibration curve to calculate the total carbohydrate content of each extract.

### 3.5. Determination of Protein

The analytical method adapted from reference [38] was modified as follows. Fifty microliters of polysaccharide extracted from TF at 0.01% (*w*/*v*) were mixed with 200 μL of BCA reagent in a 1.5 mL microtube tube. The mixture was incubated in the dark for 10 min. Following this, 200 μL of the reaction mixture was transferred to a 96-well plate, and absorbance was measured at 565 nm using a microplate reader (M200PRO, TECAN, Hsinchu, Taiwan). To determine protein content, bovine serum albumin was employed as a standard to construct a calibration curve for quantifying the protein concentration.

### 3.6. Preparation of TFP Grafted with GA

An amount of 0.2 g of TFP was dissolved in 60 mL water. GA was dissolved in 80 mL water, and an appropriate amount of EDC/NHS (as specified in the Table 2) was dissolved in 20 mL water, ensuring complete dissolution. The GA solution and EDC/NHS solution were added dropwise to the TFP solution while the pH was adjusted to the desired values. The mixture was reacted at 400 rpm and 4 °C for 1 d. After the reaction, the solution was transferred to a dialysis tube and dialyzed as described previously. The product was then freeze-dried to obtain TFP-GA.

### 3.7. Determination of Grafting Ratio of TFP-GA

The analytical method, adapted from reference [39], was conducted as follows: A 100 μL aliquot of TFP-GA (0.5%, *w*/*v*) was mixed with 100 μL of 1 N Folin–Ciocalteu reagent in a 1.5 mL microtube. The mixture was incubated in the dark for 5 min. After incubation, 1 mL of 2% sodium carbonate solution was added, and the reaction was allowed to proceed for an additional 30 min. Following this, 200 μL of the reaction mixture was transferred to a 96-well plate, and the absorbance was measured at 750 nm. GA was used as a standard, with concentrations ranging from 0 to 0.1 mg/mL, to construct a standard curve. The amount of GA in the samples was determined by comparing their absorbance values to the standard curve. The grafting ratio of TFP-GA was then calculated by dividing the amount of GA by the total weight of the sample and multiplying by 100.

### 3.8. NMR Analysis

TFP, GA, and TFP-GA were dissolved in deuterium oxide (D_2_O). The proton nuclear magnetic resonance (^1^H-NMR) spectra of these samples were analyzed using an NMR spectrometer (Agilent DD2, 600 MHz, Agilent Technologies, Inc., Santa Clara, CA, USA).

### 3.9. Scavenging Ability Against DPPH Radicals

A 40 μL aliquot of DPPH in methanol (0.8 mM) was added to a 1.5 mL microtube. To this, 180 μL of the sample at various concentrations was added, and the mixture was thoroughly mixed. The reaction was allowed to proceed in the dark at room temperature for 30 min. Following the reaction, 200 μL of the solution was transferred to a 96-well plate, and the absorbance was measured at 517 nm. The DPPH scavenging activity was calculated using the following formula:

Scavenging activity (%) = [(Ac − As)/Ac] × 100,

where Ac is the absorbance of the control and As is the absorbance of the sample. The concentration required to inhibit 50% of oxidation (IC_50_) was determined using the regression curve of scavenging activity by the sample.

### 3.10. Hydrogen Peroxide Scavenging Activity Assay

A 200 μL aliquot of a freshly prepared 20 mM solution of 30% H_2_O_2_ was added to a 1.5 mL microtube. To this, 200 μL of the sample at varied concentrations was introduced and mixed thoroughly. The reaction was incubated in the dark at room temperature for 10 min. Following the incubation, 350 μL of the reaction mixture was transferred to a quartz cuvette. Absorbance was then measured at 230 nm [40] using a spectrophotometer. Scavenging activity was assessed using the following formula:

Scavenging activity (%) = [(Ac − As)/Ac] × 100,

where Ac is the absorbance of the control and As is the absorbance of the sample.

### 3.11. Analysis of Ferrous Ion Chelating Activity

A 200 μL aliquot of the sample at different concentrations was mixed with 60 μL of a 0.18 mM ferrous chloride solution in a 1.5 mL microtube. The mixture was incubated in the dark at 37 °C for 1 h. Following the incubation, 60 μL of a 0.72 mM Ferrozine solution was added, and the mixture was thoroughly mixed and allowed to react in the dark at 37 °C for an additional 5 min. The reaction was then centrifuged at 8000 rpm for 10 min. Subsequently, 200 μL of the supernatant was transferred to a 96-well plate, and absorbance was measured at 562 nm [41] using a microplate reader. The chelating activity of ferrous ions was determined using the following equation:

Chelating activity (%) = [(Ac − As)/Ac] × 100,

where Ac is the absorbance of the control and As is the absorbance of the sample.

### 3.12. Ferric Ion Reducing Power Assay

The 100 μL sample at 0.5% (*w*/*v*) was combined with 100 μL of 0.2 M phosphate-buffered saline at pH 6.6 and 100 μL of a 1% potassium ferricyanide solution in a 1.5 mL microtube. The mixture was incubated at 50 °C for 20 min and then rapidly cooled for 5 min. Next, 100 μL of a 10% trichloroacetic acid solution was added, and the mixture was centrifuged at 4000 rpm for 5 min. A 100 μL aliquot of the supernatant was then mixed with 100 μL of double-deionized water and 30 μL of a 0.1% ferric chloride solution, allowing it to react in the dark for 10 min. Subsequently, 200 μL of this solution was transferred to a 96-well plate, and absorbance was measured at 700 nm [30]. GA and ascorbic acid, each at a concentration range of 0 to 0.1 mg/mL, were used as standards for comparison. The greater the absorbance, the higher the reducing power.

### 3.13. Determination of Nitric Oxide Scavenging Activity

A 100 μL aliquot of the sample at various concentrations was mixed with 100 μL of 10 mM sodium nitroprusside in phosphate-buffered saline in a 1.5 mL microtube. The mixture was incubated in the dark for 2 h. After incubation, 100 μL of Griess reagent was added, and the mixture was thoroughly mixed. The Griess reagent was composed of 1% (*w*/*v*) sulfanilamide in 5% phosphoric acid and 0.1% (*w*/*v*) N-(1-Naphthyl) ethylenediamine in deionized water, combined in equal volumes [42]. Subsequently, 200 μL of the reaction mixture was transferred to a 96-well plate, and absorbance was measured at 570 nm. The nitric oxide scavenging activity was calculated using the following equation:

Nitric oxide scavenging activity (%) = [(Ac − As)/Ac] × 100,

where Ac is the absorbance of the control and As is the absorbance of the sample.

### 3.14. Preparation of TFP-GA with Zinc Ions

The method was adapted with modifications from reference [43]. A 0.1 g aliquot of TFP-GA-6 was dissolved in 50 mL of water. To this solution, 1 mL of zinc nitrate solution was added in varying concentrations (80, 120, 160, and 200 mM), and the mixture was thoroughly mixed. The solution was then allowed to react in the dark at room temperature for 3 d. After the reaction, the solution was precipitated by adding ethanol to achieve a final concentration of 70%. The precipitate was collected by centrifugation at 5000 rpm for 10 min at 4 °C. The resulting precipitate was re-dissolved in water and freeze-dried using a high-efficiency freeze dryer (FDU-2200, EYELA, Tokyo, Japan) to obtain TFP-GA-Zn.

### 3.15. Antimicrobial Testing of TFP-GA-Zn

An amount of 0.1 g of TFP-GA-Zn was dissolved in water to a final volume of 5 mL, resulting in an initial concentration of 0.5% (*w*/*v*). The antimicrobial activity was tested against *S. aureus* and *E. coli*. Both bacterial strains were cultured in nutrient broth (NB) at 37 °C for 1 d. The optical density (OD) of the bacterial cultures was measured at 600 nm. The cultures were then adjusted to an OD of 1 using sterilized NB medium. Bacterial suspensions were diluted to approximate concentrations of 10^2^–10^3^ CFU/mL. For the assay, sample solutions were prepared with concentrations ranging from 0.625 to 4 mg/mL. An equal volume of each sample solution was mixed with the bacterial suspension in 1.5 mL microtubes. The mixtures were plated onto agar plates and incubated at 37 °C for 2 d. After incubation, the plates were examined visually to determine bacterial growth. If no visible growth was observed, the sample concentration was deemed bactericidal. The lowest concentration that completely inhibited visible bacterial growth was recorded as the minimal bactericidal concentration.

### 3.16. Statistical Analysis

Data were statistically analyzed using analysis of variance (ANOVA) with Statistica^TM^, (TIBCO, Palo Alto, CA, USA). Mean values were compared using Duncan’s multiple range test, with significance determined at *p* < 0.05.

## 4. Conclusions

This study compares the thermal extraction of TFP using neutral and alkaline aqueous solutions, with the latter yielding a higher amount of polysaccharides. TFP was modified with GA via the EDC/NHS-catalyzed method. A higher EDC-to-NHS ratio was found to favor the grafting reaction, while a higher pH also benefited the synthesis of TFP-GA. Increasing the amount of GA resulted in higher grafting ratios; however, excessive GA decreased the overall yield. TFP-GA exhibited strong antioxidant activity, effectively scavenging DPPH radicals, nitric oxide, and hydrogen peroxide. Additionally, TFP-GA demonstrated superior reducing ability toward Fe^3+^ and enhanced chelating activity for Fe^2+^ compared to unmodified TFP. Notably, the TFP-GA complex showed better Fe^2+^-chelating ability than GA alone. When complexed with Zn^2+^, TFP-GA displayed significantly greater antimicrobial activity against *S. aureus* and *E. coli* than TFP-GA alone, although its antioxidant activity was somewhat reduced due to chemical or steric interference from Zn^2+^. Overall, this study provides valuable insights into modifying TFP with GA via the EDC/NHS-catalyzed method. The modified TFP demonstrates both antioxidant and antimicrobial properties, making it suitable as a stabilizer in O/W emulsions to protect oxidatively labile compounds. Additionally, it can serve as a coating material for encapsulating functional ingredients in food, cosmetics, or pharmaceuticals.

## Figures and Tables

**Table 1:** Extraction, yield, and compositional analysis of *Tremella fuciformis* under neutral and alkaline conditions.

Items	Neutral (%)	Alkaline (%)
Yield	0.1 ± 0	33.9 ± 0.3
Sugars	64 ± 1	81 ± 5
Proteins	5.2 ± 0	1.3 ± 0.1
Polyphenols	0.3 ± 0	0.1 ± 0

**Table 2:** Reaction conditions, yield and grafting ratios for syntheses of TFP-GA.

Sample	EDC (mmol)	NHS (mmol)	GA (mmol)	pH	Yield (%) ^2^	Grafting Ratio (%) ^3^
TFP-GA-1	3.12	1.74	2.4	7	40.0 ± 0.4	6.3 ± 0.2 ^c 1^
TFP-GA-2	3.12	1.74	2.4	6	34.5 ± 0.1	4.7 ± 0.1 ^e^
TFP-GA-3	3.12	1.74	2.4	5	34.1 ± 0.1	3.1 ± 0.1 ^f^
TFP-GA-4	3.12	1.74	2.4	4.5	32.0 ± 0.3	2.0 ± 0 ^g^
TFP-GA-5	3.12	3.12	2.4	7	39.0 ± 0.1	5.4 ± 0.1 ^d^
TFP-GA-6	3.12	1.74	3.6	7	30.3 ± 0.3	7.7 ± 0 ^b^
TFP-GA-7	3.12	1.74	4.8	7	26.8 ± 0	8.8 ± 0.1 ^a^

^1^ Different superscripts in the same column indicate a significant difference at *p* < 0.05. ^2^ Yield is equal to [TFP-GA (wt)/((TFP (wt) + GA (wt))] × 100. ^3^ Grafting ratios were calculated from the total-phenol content in the product.

**Table 3:** Antioxidant activities of TFP-GA by different assays.

Sample	DPPH (IC_50_^1^, μg/mL)	H_2_O_2_ (IC_50_, μg/mL)	Fe^2+^ Chelation (IC_50_, μg/mL)	Vit C Equivalent ^3^ (μg/mL) ^3^	GA Equivalent ^3^ (μg/mL)	NO (IC_50_, μg/mL)
TFP	>5000	>5000	>5000	-	-	>5000
GA	45 ± 2	135 ± 1	>5000	-	-	14.3 ± 0.2
TFP-GA-1	592 ± 3 ^c 2^	698 ± 37 ^b^	685 ± 11 ^b^	588 ± 17 ^c^	197 ± 6 ^c^	250 ± 9 ^b^
TFP-GA-2	906 ± 8 ^e^	927 ± 68 ^d^	1243 ± 21 ^d^	547 ± 18 ^e^	183 ± 6 ^e^	855 ± 16 ^c^
TFP-GA-3	1354 ± 26 ^f^	>5000 ^e^	1901 ± 54 ^e^	328 ± 1 ^f^	110 ± 0 ^f^	1630 ± 51 ^d^
TFP-GA-4	3531 ± 2 ^g^	>5000 ^e^	>5000 ^f^	252 ± 2 ^g^	84 ± 1 ^g^	2200 ± 141 ^e^
TFP-GA-5	618 ± 5 ^d^	827 ± 25 ^c^	1024 ± 18 ^c^	527 ± 5 ^d^	175 ± 2 ^d^	263 ± 7 ^b^
TFP-GA-6	485 ± 7 ^b^	633 ± 7 ^a^	476 ± 15 ^a^	607 ± 1 ^b^	203 ± 0 ^b^	128 ± 1 ^a^
TFP-GA-7	361 ± 16 ^a^	586 ± 10 ^a^	463 ± 10 ^a^	631 ± 4 ^a^	212 ± 2 ^a^	113 ± 2 ^a^
TFP-GA-6-Zn	616 ± 1	786 ± 5	810 ± 24	517 ± 4	172 ± 1	257 ± 9

^1^ Concentration required to inhibit 50% of oxidation. ^2^ Different superscripts in the same column indicate significant difference at *p* < 0.05. ^3^ Ascorbic acid or gallic acid equivalent of TFP-GA and TFP-GA-Zn at 0.5% (*w*/*v*).

**Table 4:** Antimicrobial activities of TFP, TFP-GA, and TFP-GA-Zn.

Sample	*E. coli* (mg/mL) ^a^	*S. aureus* (mg/mL)
TFP	>4	>4
TFP-GA	>4	>4
TFP-GA-Zn	1.25	2.5

^a^ Minimum bactericidal concentration.

## Data Availability

Data are contained within the article.
